# The safety and efficacy of extended use of an oral shape-shifting superabsorbent hydrogel capsule for weight loss: The ELECT extension study

**DOI:** 10.1016/j.obpill.2025.100216

**Published:** 2025-10-20

**Authors:** Moshe Kamar, Donna H. Ryan, Sharon Leonard, Holly R. Wyatt, Yael Kenan, Liora Cohen Asaraf, Eti Ganon-Elazar, Jamy D. Ard

**Affiliations:** a Department of Acute Care Surgery, Edith Wolfson MC, 62 Halochhamim St. Holon, 8100, Israel; b Pennington Biomedical Research Center, Louisiana State University, 6400 Perkins Road, Baton Rouge, LA, 70808, USA; c Department of Psychiatry, Perelman School of Medicine at the University of Pennsylvania, 3535 Market St., 3rd floor, Ste #3030, Philadelphia, PA, 19104, USA; d Department of Nutrition Sciences, The University of Alabama at Birmingham, 1675 University Blvd, Birmingham, AL, 35233, USA; e Epitomee Medical Ltd, Hatochen St. Caesarea Business Park, POB 3088, Caesarea, 3079892, Israel; f Department of Epidemiology and Prevention and Department of Medicine, Wake Forest University School of Medicine, 525 Vine Street, 5th Floor, Suite 5119, Winston-Salem, North Carolina (NC), 27101, USA

**Keywords:** Maintenance, Weight loss, Hydrogel, Medical device, Obesity

## Abstract

**Background:**

Long-term weight-loss maintenance remains challenging despite of the numerous available treatments. The previously published pivotal randomized RESET study demonstrated a mean weight reduction from baseline of 6.6 % with Epitomee and a mean weight reduction of 4.6 % with placebo (p < 0.001). In this study we investigated the efficacy and safety of the extended use of Epitomee, an FDA cleared, non-pharmacological treatment for weight management.

**Methods:**

In this open label extension study, a subset of participants in the RESET study who lost ≥3 % of initial body weight during a 24-week treatment with Epitomee capsule or placebo, as an adjunct to lifestyle counseling, were eligible for a 24-week open-label extension study (ELECT).

**Results:**

Continuous treatment with Epitomee and lifestyle counseling for 48 weeks was associated with sustained reduction in body weight of 11.2 ± 8.4 % (p < 0.001) and sustained prior improvements in cardiometabolic risk factors and quality of life (QOL). Taking the Epitomee capsule for 24 weeks, after previous weight loss with the placebo treatment, was associated with an additional 1.5 ± 4.2 % weight loss, yielding a total 7.5 ± 5.5 % reduction at 48 weeks and additional improvements in QOL. Capsule adherence exceeded 94 %. Extended use of Epitomee had a favorable safety profile, with no serious adverse device-related events and no discontinuation due to adverse events.

**Conclusions:**

In this open-label study, extended use of Epitomee, combined with lifestyle counseling, was associated with maintenance of prior weight loss and favorable safety profile (NCT04994769).

## Graphical abstract

**Figure:**
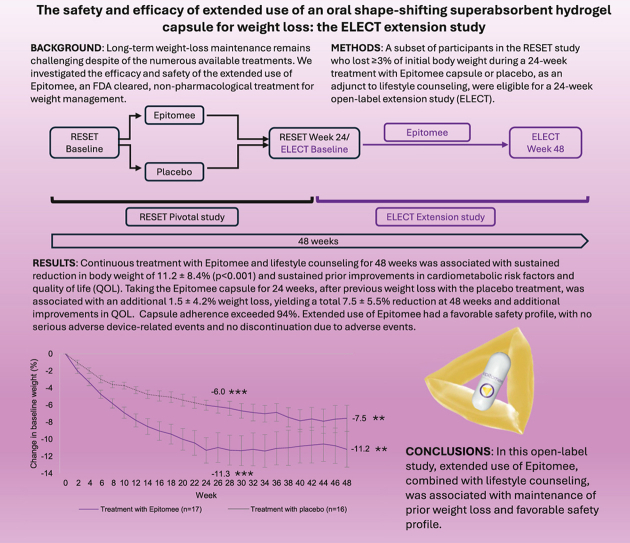

## 1 Background

The global prevalence of obesity has increased significantly over the past four decades, reaching pandemic proportions [1], [2]. Although effective treatment options for obesity are available, including lifestyle, pharmacological, and surgical interventions [3], weight regain is common after weight loss. Dietary counseling interventions have been shown to produce weight loss that diminishes over time. A review of controlled trials comparing dietary counseling-based weight-loss programs to usual care interventions found a mean net treatment effect for the former of approximately 2 body mass index (BMI) units of weight loss at 1 year (equivalent to 6 % of initial body weight or about 5 kg). However, roughly half of this initial weight loss was typically regained within 3 years [4]. In the Look AHEAD study, participants in the intensive lifestyle counseling arm lost an average of 8.6 % of their initial body weight in the first year, which decreased to 4.4 % at 4 years, below clinically significant weight loss, usually defined as a ≥5 % reduction [5]. Similar trend was reported in the Diabetes Prevention Program (DPP) trial [6], where participants in the intensive lifestyle intervention arm lost an average of approximately 7 % of their initial body weight in the first year, which decreased to about 4 % after four years. Moreover, after bariatric surgery, approximately 25 % of the initial weight loss is typically regained over a period of 6–10 years [7,8].

Weight regain has also been reported with pharmacological interventions. For example, participants who lost approximately 15 % of baseline body weight with semaglutide 2.4 mg, regained two-thirds of this loss in the year after discontinuing medication [9]. The weight-loss benefits of medications, thus, depend on continued usage; however, long-term adherence in a real-world setting is challenging due to high costs, periodic shortages, and adverse gastrointestinal events which can be intolerable in some patients [10]. Weight regain during treatment or after treatment discontinuation remains a challenge, and effective methods for weight maintenance are required.

The Epitomee capsule, a novel, FDA-cleared, minimally invasive, drug-free, orally self-administered medical device for weight management in patients with overweight or obesity, has recently been proposed as an effective tool for weight management [11,12]. The capsule is composed of absorbent polymers and bonding materials that self-expand in the stomach to create a gel-based space-occupying structure that resists the stomach's peristaltic waves. It is hypothesized to activate sensory mechanoreceptors and the gut–brain axis signaling pathway to promote early satiety, before dissolving and being excreted via the gastrointestinal tract.

In the RESET study, a prospective, randomized, double-blind, placebo-controlled trial [11], participants treated with the Epitomee capsule for 24 weeks achieved a clinically meaningful mean reduction in baseline body weight of 6.6 % [6.5] (mean [standard deviation, SD]) compared with 4.6 % [4.7] for the placebo group (p < 0.001). Of Epitomee-treated participants, 56 % attained a ≥5 % reduction in baseline body weight at 24 weeks [11]. Moreover, early response to Epitomee was associated with greater weight loss at 24 weeks compared to placebo (9.3 ± 6.0 % vs. 6.9 ± 4.3 %; p < 0.0001) [13]. Weight loss with the Epitomee capsule was found to be correlated with early satiety, decreased snacking, and reduced meal size [14], and was found to be accompanied by improvements in cardiometabolic risk factors, including waist circumference, systolic blood pressure (SBP), diastolic blood pressure (DBP), and triglyceride levels [12,14,15]. In addition, in the RESET trial, self-reported IWQOL-Lite-CT outcomes demonstrated significant improvements in physical functioning and total quality of life (QOL) scores among participants with overweight and obesity treated with Epitomee compared with placebo over 24 weeks [16]. The Epitomee capsule had a favorable safety and tolerability profile with no serious adverse device-related events [11,12].

In the present study, a subset of participants in the RESET trial, who had lost at least 3 % from baseline weight during 24 weeks of treatment with the Epitomee capsule or with placebo, both as an adjunct to lifestyle counseling, were eligible to participate in the open-label ELECT 24-week extension study (**E**xtended effect on weight **L**oss with **E**pitomee **C**apsule **T**rial). The extension study aimed to evaluate the safety and efficacy of prolonged use of the Epitomee capsule, as well as its potential effects on maintaining weight loss.

## 2 Methods

### 2.1 Study design and procedures

ELECT study is a 24-week open-label, single-arm, extension study offered to subset of participants completing the randomized, double-blind, placebo-controlled RESET study [11]. ELECT was conducted in three out of the nine sites in the United States that participated in the RESET study. These three sites were selected based on factors such as resource availability (e.g., manpower), and the ability to enroll participants at the time ELECT study was approved to be initiated.

Participants in ELECT had completed 24 weeks of treatment in RESET, with either the Epitomee capsule or placebo, and were eligible to receive an additional 24-week treatment with the Epitomee capsule (Epitomee Medical, Israel). During ELECT, the Epitomee capsule was administered as an open-label treatment along with a 15-min lifestyle counseling session provided once a month (similar to the lifestyle consultation program described previously [11], except at lower frequency). The blinding to the treatment assignment during the first 24 weeks in RESET was not revealed in effect throughout the ELECT study, although treatment was open-label in ELECT. Each Epitomee capsule had to be taken with 2 cups of water, approximately 30 min before each of two main meals. Participants were also required to monitor their physical activity, record their weight using a portable scale, and track capsule intake via the study's mobile app. Participants completing the additional 24 weeks of treatment were categorized into two groups: the Continuous-Epitomee group, consisting of participants treated with the Epitomee capsule for the entire 48 weeks, and the Switched-to-Epitomee group, consisting of participants who received placebo capsule for the initial 24 weeks of RESET, followed by 24 weeks of the Epitomee capsule in the ELECT study. The full schedule of study assessments and procedures is provided in Table S1.

The study was conducted in accordance with ICH E6, Guidelines for Good Clinical Practice, ISO 14155:2011, the US Codes of Federal Regulations (21CFR parts 11, 50, 54, 56, 812, and 814) and the Declaration of Helsinki. Written approval was obtained from the appropriate institutional review boards at each site prior to its activation. A signed informed consent form was obtained from participants prior to performing any study-related activities or evaluations.

### 2.2 Patients

The key inclusion criteria for the ELECT study were adult (≥18 years of age) participants with normoglycemia or prediabetes, and who completed 24 weeks of treatment in RESET (Epitomee capsule or placebo) [11] and lost ≥3 % of their body weight from baseline.

Prediabetes was defined as one or more of the following [1]: Fasting Plasma Glucose (FPG): A fasting blood sugar level from 100 to 125 mg/dL (5.6–6.9 mmol/L) inclusive [2] Glycated Hemoglobin (A1C): An A1C level from 5.7 % to 6.4 % (39–46 mmol/mol) inclusive.

**Fig. 1:**
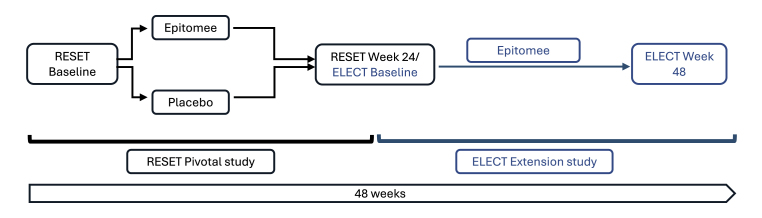
Schematic of the RESET and ELECT study designs.

The comprehensive list of inclusion and exclusion criteria can be found on clinicaltrials.gov (NCT04994769). A schematic of the RESET and ELECT study designs is presented in Fig. 1.

### 2.3 Efficacy evaluation

ELECT's objective was to evaluate the efficacy of the extended use of the Epitomee capsule, combined with lifestyle consultation, over an additional 24 weeks of treatment. In addition, we assessed the effects of the Epitomee capsule in participants who were originally assigned to placebo and lifestyle counseling in the RESET study. Only participants who lost ≥3 % of their body weight from baseline during the RESET study were considered eligible. The primary endpoint was the percent change in body weight after 48 weeks of treatment, as measured from baseline in RESET to week 48 in ELECT. Additional analyses of secondary and exploratory endpoints presented here included: the proportion of participants with a reduction of ≥5 %, ≥7 % and ≥10 % of baseline body weight at week 48; change in BMI classification; changes in mean SBP and DBP; change in laboratory values [triglycerides, low-density lipoprotein cholesterol (LDL-C), high-density lipoprotein cholesterol (HDL-C), fasting serum insulin, fasting plasma glucose (FPG) and hemoglobin A1C (HbA1C)]; change in waist circumference; and change in quality of life (QOL) measured using the Impact of Weight on Quality of Life-Lite-Clinical Trials (IWQOL-Lite-CT) questionnaire [17,18] at week 24 and week 48.

### 2.4 Safety assessment

The safety and tolerability of the extended use of the Epitomee capsule (48-week treatment) were assessed as well. The primary safety endpoint was the incidence of serious adverse device-related events from RESET's baseline assessment (week 24) through week 48. Secondary safety endpoints included incidence of all adverse events coded according to the Medical Dictionary for Regulatory Activities (MedDRA).

### 2.5 Statistics

Descriptive statistics, including mean ± SD for continuous variables and proportions for categorical variables, were calculated. Comparative analyses of continuous variables, including change from baseline in body weight (% reduction), waist circumference, SBP, DBP and QOL were conducted between groups by Student's t-test and within groups by paired *t*-test. Comparative analyses of categorical variables were conducted by Chi-squared test or Fisher's exact test and summarized using number of observations and percentages. Safety data were examined as observed proportions or means. Statistical significance was defined as p < 0.05. The analyses presented in this study were conducted on observed data only. To present the weight-loss trajectory, missing data were addressed using interpolation, in which missing values were estimated by calculating the average of the weight-loss values from the preceding and subsequent weeks.

Statistical analyses were performed using SAS® Version 9.4 Windows® 2008 Terminal and Python® Version 3.9, employing Statistical models and Sklearn modules for statistical calculations.

## 3 Results

### 3.1 Study participants

The ELECT study was offered to 55 participants who had completed the RESET study (at three sites), had lost ≥3 % body weight from baseline at week 24, and who met other eligibility criteria. Of these participants, 33 signed the informed consent and were enrolled: 17 to the Continuous-Epitomee group and 16 to the Switched-to-Epitomee group (Fig. S1). Of 55 participants who met the eligibility criteria, 22 chose not to participate in ELECT. Baseline demographics for those 22 participants are provided in Table S2.

Overall, 4 participants (12.1 %) discontinued treatment during the study; 1 participant in the Continuous-Epitomee group due to non-compliance and 3 in the Switched-to-Epitomee group due to traveling or pregnancy. None of the withdrawals from treatment were due to adverse events (Fig. S1).

Table 1 displays baseline characteristics (gender age, and race) of the two groups.

**Table 1:** Summary of demographics and baseline characteristics at baseline.

	Continuous Epitomee^a^ (n = 17)	Switched to Epitomee^b^ (n = 16)	Difference (95 % CI)
Age (years), mean ± SD (N)	52.4 ± 8.8 (17)	49.6 ± 14.4 (16)	2.8 (−5.4,11.0)

Gender, % (n/N)			
Female	82.4 (14/17)	81.2 (13/16)	1.1
Male	17.6 (3/17)	18.8 (3/16)	−1.1

Race/Ethnicity, % (n/N)			
White	64.7 (11/17)	87.5 (14/16)	−22.8
Black or African American	23.5 (4/17)	6.2 (1/16)	17.3
Hispanic/Latino	0.0 (0/17)	0.0 (0/16)	0.0
Asian	0.0 (0/17)	6.2 (1/16)	−6.2
American Indian or Alaska Native	5.9 (1/17)	0.0 (0/16)	5.9
Multiple race	5.9 (1/17)	0.0 (0/16)	5.9

Abbreviations: CI, confidence Interval; SD, standard deviation.

a Continuous Epitomee group: participants who received Epitomee capsule for the initial 24 weeks of RESET, followed by 24 weeks of the Epitomee capsule in the ELECT study (total of 48 weeks treatment with Epitomee capsule).

b Switched-to-Epitomee group: participants who received placebo capsule for the initial 24 weeks of RESET, followed by 24 weeks of the Epitomee capsule in the ELECT study.

At entry to the ELECT study participants who were treated with Epitomee for 24 weeks had exhibited numerically lower BMI and FPG compared to those treated with placebo. BMI categories also differed between these two groups, which can be attributed to the greater weight loss achieved by participants treated with Epitomee during RESET. Weight and other characteristics at baseline and at time of enrollment in the ELECT study (i.e., at week 24) are provided in Table S3.

Capsule adherence during the ELECT study was high for both groups, with participants in the Continuous-Epitomee group taking 94.1 % of the expected capsules and participants in the Switched-to-Epitomee group taking 98.1 % of capsules over the additional 24 weeks of treatment.

### 3.2 Assessment of percent reduction in baseline body weight at week 48

At entry into the ELECT study (week 24), participants treated with Epitomee and lifestyle counseling had lost 11.3 ± 6.2 % of baseline body weight. With 24 additional weeks of Epitomee and lifestyle counseling, these participants maintained a loss of 11.2 ± 8.4 % of baseline body weight at week 48 (*p* < 0.001 relative to the RESET baseline).

**Fig. 2:**
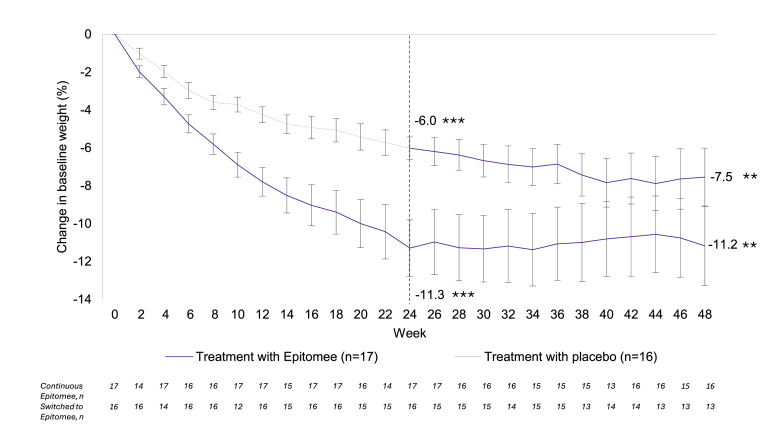
Percent change in body weight from baseline over 48 weeks, in participants receiving continuous Epitomee or in participants receiving placebo for 24 weeks followed by Epitomee for 48 weeks. ∗∗P < 0.001; ∗∗∗P < 0.0001.

Participants originally treated with placebo and lifestyle counseling in the RESET study and who switched to Epitomee, lost on average 6.0 ± 2.4 % of baseline weight at week 24. With the addition of the Epitomee capsule during the subsequent 24 weeks of the ELECT study, these participants lost an additional 1.5 ± 4.2 % body weight between weeks 24–48 (p < 0.2728). They achieved a total weight loss of 7.5 ± 5.5 % at week 48 (*p* < 0.001 relative to the RESET baseline) (Fig. 2).

### 3.3 Effect of 48 weeks treatment with epitomee on weight, cardiometabolic parameters and QOL parameters

In the Continuous-Epitomee group, 75 % of the participants who completed 48 weeks of treatment with Epitomee and lifestyle counseling attained ≥5 % reduction, 62.5 % achieved ≥7 % reduction, and 37.5 % attained ≥10 % reduction in baseline bodyweight (Fig. S2).

In addition, participants in the Continuous-Epitomee group significantly improved their waist circumference, SBP and IWQOL Lite CT scores at week 24, improvements that were maintained through week 48. DBP remained within normal ranges throughout the entire Epitomee treatment period (Table 2). Participants who attained ≥10 % weight reduction at week 24 showed greater improvements at week 48 in psychosocial and total scores (Table 2).

**Table 2:** Effect of 48 weeks of continuous Epitomee treatment on cardiometabolic risk factors and QOL.

	Baseline Mean ± SD (N)	Week 24 Mean ± SD (N)	Week 48 Mean ± SD (N)
**Waist circumference, cm**	107.9 ± 11.5 (16)	97.1 ± 8.6 (16)^a^	96.9 ± 11.3 (16)^a^

**SBP, mmHg**	127.2 ± 15.2 (16)	120.7 ± 14.7 (16)^a^	121.5 ± 19.1 (16)

**DBP, mmHg**	76.3 ± 12.1 (16)	73.8 ± 9.7 (16)	76.6 ± 10.6 (16)

**IWQOL [Total study population]**			
Physical	66.1 ± 16.8 (16)	77.2 ± 15.4 (16)^a^	75.0 ± 16.3 (16)^a^
Physical Function	66.6 ± 20.9 (16)	80.9 ± 15.8 (16)^a^	77.8 ± 16.9 (16)^a^
Psychosocial	52.5 ± 18.8 (16)	67.7 ± 17.0 (16)^a^	71.8 ± 17.6 (16)^a^
Total	57.9 ± 17.4 (16)	71.0 ± 15.9 (16)^a^	72.9 ± 15.6 (16)^a^

**IWQOL [≥10 % weight loss at week 24]**			
Physical	63.9 ± 12.6 (8)	76.4 ± 11.0 (8)^a^	73.2 ± 12.1 (8)
Physical Function	63.8 ± 18.7 (8)	80.6 ± 11.2 (8)^a^	76.3 ± 7.9 (8)
Psychosocial	54.1 ± 15.2 (8)	70.0 ± 14.0 (8)^a^	79.1 ± 9.9 (8)^a^
Total	57.5 ± 13.1 (8)	72.2 ± 12.3 (8)^a^	77.0 ± 8.1 (8)^a^

**Labs**			
HDL-C (mg/dL) Normal range: ≥60 mg/dL	56.6 ± 12.8 (16)	57.6 ± 12.7 (16)	61.2 ± 13.7 (16)^a^
LDL-C (mg/dL) Normal range: ≤130 mg/dL	128.4 ± 29.1 (16)	124.0 ± 36.8 (16)	116.5 ± 24.5 (16)
Triglycerides (mg/dL) Normal range: ≤150 mg/dL	105.5 ± 49.2 (16)	87.9 ± 32.3 (16)^a^	88.9 ± 39.9 (16)
Serum insulin (μIU/mL) Normal range: ≤20 μIU/mL	13.7 ± 8.4 (16)	6.9 ± 4.6 (15)^a^	9.4 ± 3.4 (16)^a^
HbA1c (%) Normal range: ≤5.7 %	5.6 ± 0.4 (16)	5.5 ± 0.3 (15)	5.5 ± 0.4 (16)
Fasting glucose (mg/dL) Normal range: ≤100 mg/dL	90.8 ± 15.5 (16)	81.9 ± 8.7 (15)	89.2 ± 9.8 (16)

Abbreviations: DBP, diastolic blood pressure; HbA1c, glycated hemoglobin; HDL, high-density lipoprotein; IWQOL, Impact of Weight on Quality of Life-Lite-Clinical Trials; LDL, low-density lipoprotein; SD, standard deviation; SBP, systolic blood pressure.

a Significant change from baseline.

Overall, the average laboratory values of HDL-C, LDL-C, triglycerides, serum insulin, HbA1C and FPG were relatively stable during 48 weeks of treatment, remaining within or close to normal ranges. HDL-C levels for participants treated with Epitomee for 48 weeks reached 61.2 ± 13.7 mg/dl by week 48, a significant increase from RESET baseline (Table 2).

### 3.4 Effect of 24 weeks of treatment with placebo followed by 24 weeks of treatment with epitomee on weight, cardiometabolic parameters and QOL parameters

In the Switched-to-Epitomee group, 69.2 % of the participants attained ≥5 % reduction, 53.8 % attained ≥7 % reduction and 15.4 % attained ≥10 % reduction in baseline weight at week 48 (Fig. S3).

In addition, participants in the Switched-to-Epitomee group significantly improved waist circumference, SBP and DBP at week 24 and maintained these improvements at week 48 (Table 3).

**Table 3:** Effect of 24 weeks of treatment with placebo followed by 24 weeks of treatment with Epitomee on cardiometabolic risk factors and QOL.

	Baseline Mean ± SD (N)	Week 24 Mean ± SD (N)	Week 48 Mean ± SD (N)
**Waist circumference, cm**	105.7 ± 8.4 (13)	100.4 ± 9.4 (13)^a^	99.8 ± 10.9 (12)^a^

**SBP. mmHg**	124.0 ± 10.3 (13)	119.9 ± 11.9 (13)	118.8 ± 11.8 (13)

**DBP, mmHg**	82.4 ± 10.6 (13)	76.5 ± 9.4 (13)	78.1 ± 8.5 (13)

**IWQOL**			
Physical	57.1 ± 10.9 (13)	65.7 ± 16.0 (13)	72.5 ± 10.9 (13)^a^
Physical Function^b^	60.0 ± 11.7 (13)	68.5 ± 17.0 (13)^a^	77.3 ± 12.7 (13)^a^
Psychosocial	50.7 ± 17.3 (13)	61.5 ± 19.0 (13)^a^	67.2 ± 10.7 (13)^a^
Total	53.0 ± 13.7 (13)	63.0 ± 16.9 (13)^a^	69.0 ± 10.0 (13)^a^

**Labs**			
HDL-C (mg/dL) Normal range: ≥60 mg/dL	51.3 ± 11.3 (12)	55.1 ± 13.5 (13)	50.4 ± 11.6 (13)
LDL-C (mg/dL) Normal range: ≤130 mg/dL	117.8 ± 31.5 (12)	108.2 ± 18.3 (13)	103.3 ± 25.4 (13)^a^
Triglycerides (mg/dL) Normal range: ≤150 mg/dL	112.5 ± 51.0 (12)	92.4 ± 30.5 (13)^a^	112.3 ± 47.1 (13)
Serum insulin (μIU/mL) Normal range: ≤20 μIU/mL	11.4 ± 4.4 (13)	8.9 ± 5.3 (12)	10.2 ± 7.7 (12)
HbA1c (%) Normal range: ≤5.7 %	5.6 ± 0.3 (13)	5.5 ± 0.3 (12)^a^	5.6 ± 0.3 (13)
Fasting glucose (mg/dL) Normal range: ≤100 mg/dL	90.6 ± 7.9 (13)	90.0 ± 12.5 (12)	89.2 ± 6.2 (12)

Abbreviations: DBP, diastolic blood pressure; HbA1c, glycated hemoglobin; HDL, high-density lipoprotein; IWQOL, Impact of Weight on Quality of Life-Lite-Clinical Trials; LDL, low-density lipoprotein; SD, standard deviation; SBP, systolic blood pressure.

a Significant change from baseline.

b Significant change between week 24 and week 48.

These participants also demonstrated improved IWQOL scores at week 24 with notable, further improvement over the following 24 weeks, including a significant improvement in physical function (Table 3).

Overall, the average laboratory values of HDL-C, LDL-C, triglycerides, serum insulin, HbA1C and FPG were relatively stable during 48 weeks of treatment, remaining within or close to normal ranges.

### 3.5 Safety assessment

During the RESET [11] and ELECT studies, no serious adverse events were attributed to treatment with Epitomee (Table 4). Moreover, 48 weeks of treatment with Epitomee was not associated with an increase in the overall incidence of adverse events compared to 24 weeks of treatment [11]. During the ELECT study, most adverse events in the Continuous-Epitomee group were mild in severity and assessed as unrelated to the investigated product. Most adverse events were transient with 89.7 % resolved prior to study termination (Table 4). The most frequently reported adverse events in participants treated for 48 weeks with Epitomee were COVID-19, in 17.6 % of participants, and abdominal discomfort, dyspepsia and flatulence, in 11.8 % of the participants, each. Safety data for ELECT's Switched to Epitomee group (n = 16) are not displayed here, as safety data from 24 weeks treatment with Epitomee in 138 participants have been previously reported [11] and are presented in this manuscript.

**Table 4:** Adverse events reported during 24 and 48 weeks of Epitomee treatment.

	24 weeks Epitomee (RESET) (N = 138) [11]		Continuous Epitomee (ELECT) (N = 17)^a^	
	No. of participants (%)	No. of events	No. of participants (%)	No. of events
**Any adverse event**	119 (86.2)	357	13 (76.5)	29

**Seriousness**				
**Serious adverse events**^b^	1 (0.7)	1	0	0
Related serious adverse events	**None**			

**Severity**				
Mild adverse event	107 (77.5)	244	12 (70.6)	25
Moderate adverse event	51 (37.0)	97	4 (23.5)	4
Severe adverse event	12 (8.7)	16	0 (0.0)	0

**Causality**				
Related adverse event	42 (30.4)	62	5 (29.4)	6
Unrelated adverse event	111 (80.4)	295	13 (76.5)	23

**Adverse gastrointestinal events**	54 (39.1)	83	8 (47.1)	11

**Number of adverse events leading to withdrawal**	2 (1.4 %)	2	0	0

ELECT's Switched to Epitomee group (n = 16) is not displayed here. Safety data on 24 weeks treatment with Epitomee was previously reported for 138 participants [11] and is presented here.

a Assessed during ELECT (week 24 to week 48).

b Two participants experienced serious adverse event during the RESET study (first 24 weeks): one participant in the Epitomee group experienced a Helicobacter pylori–related gastric ulcer, and one participant in the placebo group experienced a transient ischemic attack. There were no device-related SAEs.

## 4 Discussion

This open-label, observational study evaluated the safety and efficacy of prolonged use of the Epitomee capsule, as well as its potential effects when transitioning to Epitomee treatment following weight loss achieved with placebo and lifestyle counseling. Participants treated with Epitomee for 24 weeks, who enrolled in the open-label extension study, achieved a mean weight loss of 11.3 % from baseline. By continuing treatment for another 24 weeks, they maintained this clinically significant weight reduction, achieving an average reduction of 11.2 % weight loss at 48 weeks. At 28 weeks and 56 weeks, liraglutide combined with lifestyle counseling resulted in maximal weight loss of 8.4 % at week 28, which was maintained at week 56 (7.4 %) [19].

While the numbers of participants were small in ELECT, there were small differences in weight loss according to whether participants had received placebo or Epitomee over the first 24 weeks of observation. Participants previously on placebo lost an additional 1.5 % of their body weight, while no further weight loss was observed in those who had been on Epitomee continuously. However, total weight loss was greater in those who received Epitomee for the full 48 weeks compared to those who started after placebo, consistent with research showing that larger initial weight loss predicts greater overall weight loss outcomes [20].

Several studies have shown that 5 %–7 % reduction in baseline body weight are associated with clinically relevant improvements in CVD risk factors [[21], [22], [23], [24], [25]], which is consistent with the results presented here.

Participants in the continuous Epitomee group showed an improvement in IWQOL-Lite-CT scale scores at week 24 and were able to maintain those improvements with continued treatment at week 48. The addition of Epitomee treatment after 24 weeks of previous weight loss with lifestyle counseling was associated with additional improvement across all measured IWQOL-Lite-CT scores, with physical function significantly improving.

The favorable safety and tolerability of the Epitomee capsule and its apparent association with fewer adverse events compared to other available treatments have been previously reported [11], with notable similarity in the rate and type of AEs between the Epitomee and placebo groups. Extended use of Epitomee capsules for 48 weeks was well tolerated, with 94.1 % capsule adherence in the Epitomee continuous group and a low dropout rate of 12 % across both groups, none due to adverse events. There were no serious adverse device-related effects.

This open-label observational study provides preliminary results suggesting that extended use of Epitomee, a non-pharmacological treatment combined with lifestyle counseling, is associated with weight loss maintenance and demonstrated a favorable safety profile.

## 5 Limitations

The ELECT study has several limitations that should be considered. These include a small sample of participants in both treatment groups, which limits the generalizability of the present findings and reduces the statistical power to detect differences between groups. Second, some of the analyses were not prespecified, and the results can only be deemed hypothesis-generating. In addition, all ELECT participants were required to lose at least 3 % of baseline body weight in the initial 24-week RESET study, whether originally assigned to Epitomee or placebo. Thus, participants in ELECT were relative treatment responders to Epitomee and/or lifestyle counseling; 48-week weight losses of these participants are likely to be larger than those that would be achieved by treatment non-responders in RESET. As the current data extend only to 48 weeks, future investigations assessing the long-term effects of Epitomee would be valuable. In addition, since Epitomee is indicated for use in combination with lifestyle counseling, future studies should also evaluate its role within broader combination therapy approaches. The study also would have been strengthened by the use of a randomized controlled design in which participants who initially lost 3 % of body weight with Epitomee were re-randomized at week 24 (in double-blind manner) to continued use of Epitomee or to placebo. This design, often used in trials of anti-obesity medications, provides stronger evidence that the effects observed (e.g., maintenance of weight loss) are attributable to the intervention and not to uncontrolled or confounding factors (e.g., participant expectation or motivation) [26]. Still, the present data provide initial evidence that Epitomee, combined with lifestyle counseling, can sustain weight loss for up to 48 weeks with high patient adherence and tolerability. Future studies that address these limitations are needed.

## 6 Conclusions

In conclusion, the ELECT open label study found that the Epitomee capsule was associated with the maintenance of weight loss, previously achieved with either Epitomee or placebo (both combined with lifestyle counseling) and with sustaining improvements in the cardiometabolic risk factors and quality of life parameters that accompanied weight loss. The addition of Epitomee after previous weight loss with placebo and lifestyle counseling was associated with small additional weight loss. Extended use of the Epitomee capsule up to 48 weeks was well-tolerated and had a favorable safety profile, with no serious adverse device-related events. The combination of weight-loss maintenance with a favorable safety profile highlights the potential role of the Epitomee capsule in weight-loss management.

### 6.1 Key takeaways

• The ELECT open label study found that the Epitomee capsule was associated with the maintenance of weight loss, previously achieved with either Epitomee or placebo (both combined with lifestyle counseling) and with sustaining improvements in the cardiometabolic risk factors and quality of life parameters that accompanied weight loss.
• Extended use of the capsule for up to 48 weeks was well tolerated, with a favorable safety profile and no serious device-related adverse events.
• This combination of weight-loss maintenance with a favorable safety profile highlights the potential role of the Epitomee capsule in weight-loss management.

## Author contribution

Moshe Kamar. contributed to conceptualization, data curation, formal analysis, writing the original draft and reviewing and editing the draft. Donna H. Ryan contributed to conceptualization, writing the original draft, and reviewing and editing the draft. Sharon Leonard contributed to the supervision of the study. Holly R. Wyatt contributed to investigation and reviewing and editing the draft. Yael Kenan contributed to conceptualization, investigation, reviewing, editing the draft and the supervision of the study. Eti Ganon-Elazar contributed to investigation, data curation, formal analysis, reviewing and editing the draft, visualization and data administration. Liora Asaraf Cohen contributed to data curation, formal analysis, reviewing and editing the draft, visualization, and data administration. Jamy D. Ard contributed to investigation and reviewing and editing the draft.

## Ethical adherence and ethical review

This prospective clinical trial involved interventions affecting human test subjects, was conducted in accordance with the Code of Ethics of the World Medical Association (Declaration of Helsinki) for experiments involving humans and received approval by independent ethics committees or institutional review boards at each study site. All participants provided written informed consent. The study protocol was registered on clinicaltrials.gov (NCT04994769).

Unless otherwise stated, responsibility for editorial decisions and peer review process for this article was delegated to non-author Editors or non-author Associate Editors.

## Declaration of artificial intelligence (AI) and AI-assisted technologies in the writing process

During the preparation of this work the authors did not use AI assisted technologies.

## Source of funding

This study was funded by Epitomee Medical Ltd.
