# Supplementary material for: The safety and efficacy of extended use of an oral shape-shifting superabsorbent hydrogel capsule for weight loss: The ELECT extension study
Source: Obes Pillars. 2025 Oct 20;16:100216. doi: 10.1016/j.obpill.2025.100216 (PMC12597288; doi:10.1016/j.obpill.2025.100216)
Supplement: Multimedia component 1 [file mmc1.docx]

Supplementary data


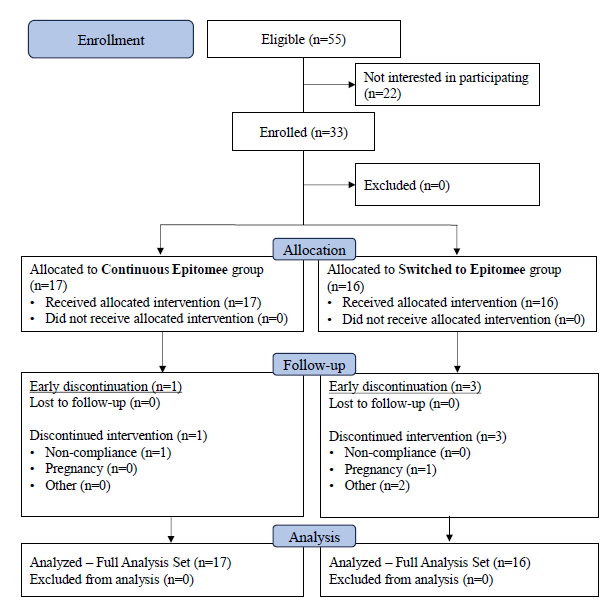


**FIGURE S1: Participant disposition**


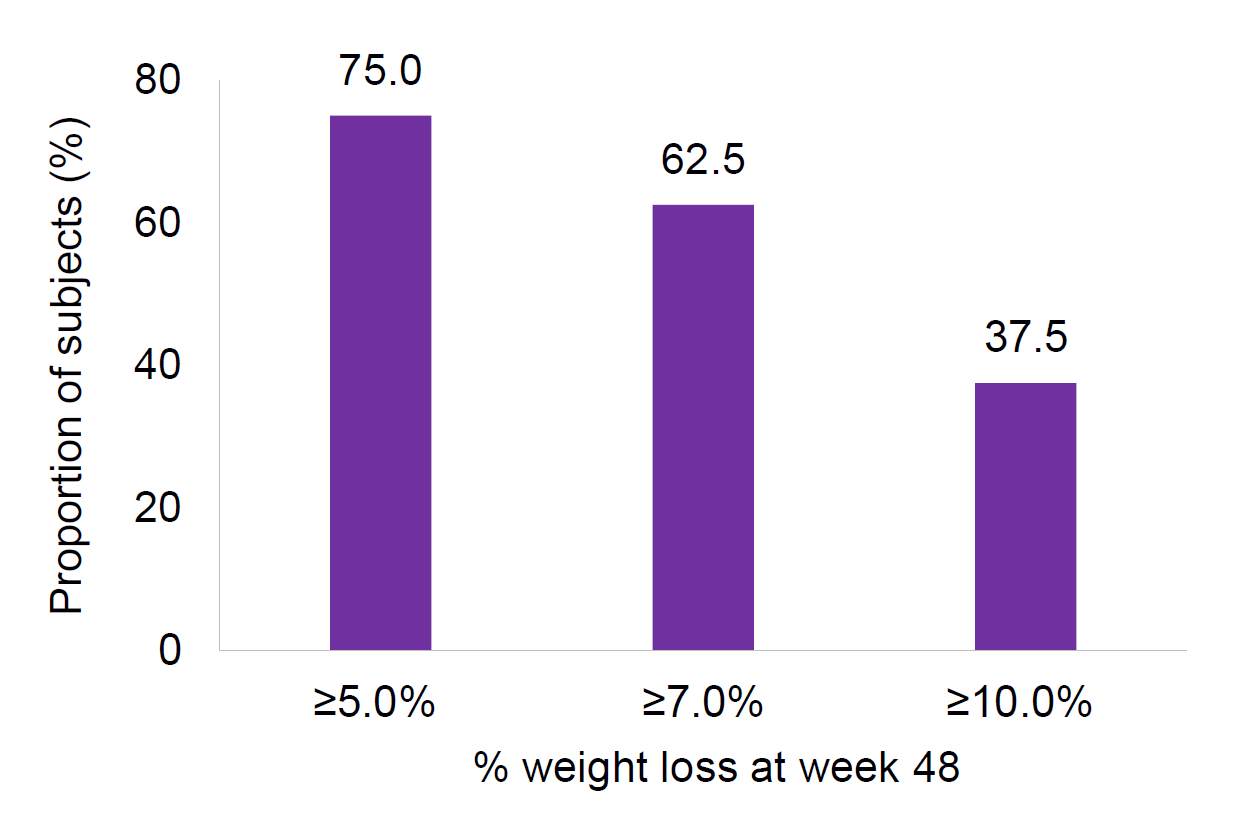


**FIGURE S2: Proportion of participants completing 48 weeks of treatment with Epitomee (n=16) with ≥5% (N = 12), ≥7% (N = 10), and ≥10% (N = 6) reduction in baseline body weight at week 48.**


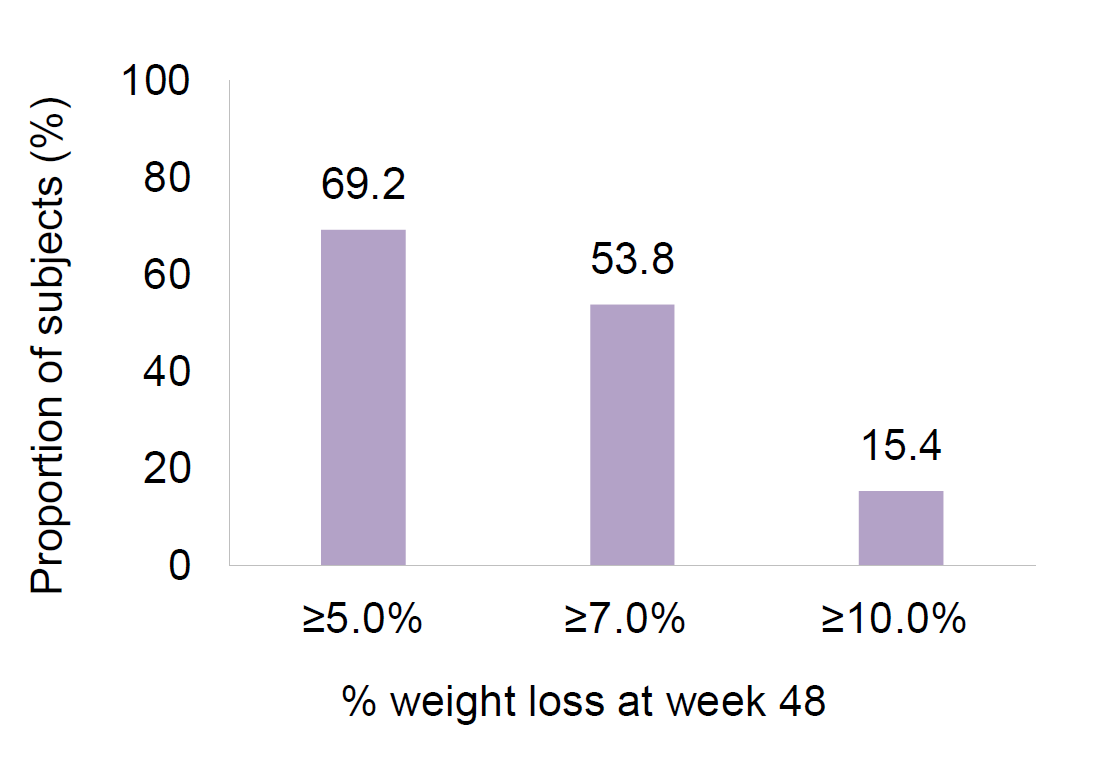


**FIGURE S3: Effect of 24 weeks treatment with placebo and lifestyle counseling followed by 24 weeks treatment with Epitomee capsule, on proportion of participants with** **≥5% (N = 9), ≥7% (N = 7), and ≥10% (N = 2) reduction in initial body weight at week 48**

| **Visit week** | **Screening visit^1,2, 3^ (also Week 24 of core study)** | **0^1^** | **2** | **4** | **6** | **8** | **10** | **12** | **14** | **16** | **18** | **20** | **22** | **24 or ET^10^** | **Unscheduled visit^4^** |
| --- | --- | --- | --- | --- | --- | --- | --- | --- | --- | --- | --- | --- | --- | --- | --- |
| **Visit Window (days)** | **-7** | **±1** | **±2** | **±2** | **±2** | **±2** | **±2** | **±2** | **±2** | **±2** | **±2** | **±2** | **±2** | **±2** |  |
| Informed consent | X |  |  |  |  |  |  |  |  |  |  |  |  |  |  |
| Medical-history confirmation | X |  |  |  |  |  |  |  |  |  |  |  |  |  |  |
| Review inclusion and exclusion | X | X |  |  |  |  |  |  |  |  |  |  |  |  |  |
| Medical monitoring/ assessment | X | X |  | X |  | X |  | X |  | X |  | X |  | X |  |
| Weight and BMI (using in -Clinic scale)**^5^** | X | X | X | X | X | X | X | X | X | X | X | X | X | X |  |
| Waist Circumference**^6^** | X |  |  |  |  |  |  | X |  |  |  |  |  | X |  |
| Vital Signs**^7^** | X | X | X | X | X | X | X | X | X | X | X | X | X | X |  |
| Lifestyle Intervention (15 min face to face)**^8^** |  | X |  | X |  | X |  | X |  | X |  | X |  | X |  |
| Quality of Life (IWQOL-CT) | X |  |  |  |  |  |  |  |  |  |  |  |  | X |  |
| Subjects’ satisfaction scale | X |  |  |  |  |  |  | X |  |  |  |  |  | X |  |
| Lab tests- **^9^** | X |  |  |  |  |  |  | X |  |  |  |  |  | X |  |
| Urinalysis | X |  |  |  |  |  |  |  |  |  |  |  |  | X |  |
| Urine pregnancy test | X | X |  |  | X |  |  | X |  |  | X |  |  | X |  |
| EKG | X |  |  |  |  |  |  |  |  |  |  |  |  | X |  |
| Capsule dispensing/accountability |  | X | X | X | X | X | X | X | X | X | X | X | X | X |  |
| Study app re-training and capsule animation video |  | X |  |  |  |  |  |  |  |  |  |  |  |  |  |
| Home Weight (at subjects’ home; at least twice a week)**^5^** |  | Throughout the study | | | | | | | | | | | | |  |
| Physical tracker and food diary |  | Throughout the study | | | | | | | | | | | | |  |
| Concomitant Medications | X | X | X | X | X | X | X | X | X | X | X | X | X | X | X |
| Adverse Events | X | X | X | X | X | X | X | X | X | X | X | X | X | X | X |
| Study sensor devices collect^11^ |  |  |  |  |  |  |  |  |  |  |  |  |  | X |  |
| **^1^Screening Visit of** this study correspond with week 24 of the core study. **Overlapping assessments will not be repeated (including EKG, Questionnaires,** weight measurements, waist **Measurements** lab tests and vital **Signs).** Final assessment to conclude eligibility of the subject will be done at week 0 (blood tests results are not available at the screening visits therefore eligibility can only be finalized in week 0 visit).  **^2^ Demographic Data and physical examination**: performed/collected on screening visit of Epitomee core study and will be used for this study.  **^3^ Height:** Subject’s height was measured at screening visit of the Epitomee core study and will be used for this study.  **^4^ Unscheduled visit** will be conducted as per PI decision to further follow up on subjects' safety or for any other reasons.  **^5^ Weight** will be measured at each visit using in clinic weight scale. In addition, subjects will use the portable connected scale (at least twice a week) throughout the study as instructed.  **^6^ Waist circumference** will be measured at the screening visit and at week 12 and 24.  **^7^ Vital Signs:** Heart Rate (HR), Systolic and diastolic blood pressures (BP) [while sitting].  **^8^ Life Style consultation** will be done once a month. Site Lifestyle personnel will review the accumulated data of the following; food and calorie intake using mobile app (or paper-and-pencil), portable weight scale measures, physical activity tracker (watch) and capsule consumption  **^9^Labs- Test: The following blood tests will be collected** (Lab Corp Ltd, is the central laboratory vendor for this study)**:**   1. **Chemistry:** Blood will be collected at fasting for comprehensive metabolic panel week at the screening visit and at week 12 and 24 (glucose, calcium, albumin, total protein, sodium, potassium, CO2, Chloride, BUN, magnesium, phosphorus, serum creatinine, ALP, ALT, AST, total bilirubin, direct bilirubin, indirect bilirubin, creatine kinase (CK), GGT, globulin, iron, LDH, uric acid). 2. **Hematology:** Blood will be collected for Hematology panel at the screening visit and at week 12 and 24 (WBC, RBC, platelets, hemoglobin, WBC, RBC, hematocrit, neutrophils) 3. **Fasting plasma glucose and hemoglobin A1C (FPG, AIC)** Blood will be collected for testing at the screening visit and at week 12 and 24. 4. **PT:** Blood will be collected for testing at the screening visit and at week 24 5. **Serum insulin:** Blood will be collected for testing at the screening visit and at week 12 and 24. 6. **Lipid profile:** Blood will be collected for testing at the screening visit and at week 12 and 24 (cholesterol, very low-density lipoprotein calculation VLDL, HDL-C, LDL-C, Triglycerides,) 7. **hs-CRP:** Blood will be collected for testing at the screening visit and at week 24.   **^10^ Early Termination Visit:** Upon premature termination, subject will perform study week 24 assessments.  **^11^ Loaned Equipment:** study sensor devices loaned at the ‘core’ study will be returned at week 24 visit. | | | | | | | | | | | | | | | |

**TABLE S1: Study assessment schedule.**

|  | **Enrolled to ELECT** | **Not interested to participate** | **Difference (95% CI)** |
| --- | --- | --- | --- |
| **Age (years), mean ± SD (N)** | 51.1 ± 11.8 (33) | 54.1 ± 13.2 (22) | -3.1 (-9.9, 3.8) |
| **Weight (kg) , mean ± SD (N)** | 94.4 ± 14.0 (33) | 97.2 ± 13.1 (22) | -2.8 (-10.1, 4.4) |
| **BMI (kg/m2) , mean ± SD (N)** | 33.5 ± 3.3 (33) | 34.6 ± 3.2 (22) | -1.2 (-2.9, 0.6) |
| **Waist (cm), mean ± SD (N)** | 107.4 ± 10.2 (33) | 111.5 ± 9.3 (22) | -4.2 (-9.4, 1.1) |
| **Gender, %** (n/N) |  |  |  |
| Female | 81.8 (27/33) | 68.2 (15/22) | 13.6 |
| Male | 18.2 (6/33) | 31.8 (7/22) | -13.6 |
| **Race or Ethnicity, %** (n/N) |  |  |  |
| White | 75.8 (25/33 | 81.8 (18/22) | -6.1 |
| Black or African American | 15.2 (5/33) | 4.5 (1/22) | 10.6 |
| Asian | 3.0 (1/33) | 9.1 (2/22) | 6.1 |
| American Indian or Alaska Native | 3.0 (1/33) | 4.5 (1/22) | -1.5 |
| **Glycemic Status, %** (n/N) |  |  |  |
| Normoglycemia | 57.6 (19/33) | 50.0 (11/22) | 7.6 |
| Prediabetes | 39.4 (13/33) | 45.5 (10/22) | -6.1 |
| Diabetes | 3.0 (1/33) | 4.5 (1/22) | -1.5 |
| 22 participants who met the eligibility criteria were not interested in participating in the ELECT study. It is noteworthy that these 22 participants were matched to the 33 participants enrolled in the ELECT study in terms of age, baseline weight, BMI, waist circumference, and glycemic status; however, they differed in gender and ethnicity.  Abbreviations: CI, confidence Interval; SD, standard deviation. | | | |

**Table S2: Summary of baseline demographics for participants enrolled in the ELECT study and participants not interested to participate.**

|  | **Continuous Epitomee** | | **Switched to Epitomee** | |
| --- | --- | --- | --- | --- |
|  | **Baseline** | **Week 24** | **Baseline** | **Week 24** |
| Weight (kg), mean ± SD (N) | 95.0 ± 15.0 (17) | 84.1 ± 12.7 (17) | 93.7 ± 13.3 (16) | 88.4 ± 12.7 (16) |
| BMI (kg/m^2^), mean ± SD (N) ^#^ | 33.1 ± 3.1 (17) | 29.3 ± 2.8 (17) | 33.9 ± 3.6 (16) | 32.0 ± 3.6 (16) |
| Waist circumference (cm), mean ± SD (N) | 108.0 ± 11.2 (17) | 97.5 ± 8.5 (17) | 106.7 ± 9.3 (16) | 102.1 ± 10.2 (16) |
| Weight categories, % (n/N) ^#^ | | | |  |
| Normal | 0.0 (0/17) | 23.5 (4/17) | 0.0 (0/16) | 6.2 (1/16) |
| Overweight | 11.8 (2/17) | 29.4 (5/17) | 12.5 (2/16) | 18.8 (3/16) |
| Obesity Class I | 58.8 (10/17) | 47.1 (8/17) | 43.8 (7/16) | 50.0 (8/16) |
| Obesity Class II | 29.4 (5/17) | 0.0 (0/17) | 43.8 (7/16) | 25.0 (4/16) |
| Glycemic status, % (n/N) | | | |  |
| Missing | 0.0 (0/17) | 5.9 (1/17) | 0.0 (0/16) | 6.2 (1/16) |
| Normoglycemia | 58.8 (10/17) | 64.7 (11/17) | 56.2 (9/16) | 62.5 (10/16) |
| Prediabetes | 35.5 (6/17) | 29.4 (5/17) | 43.8 (7/16) | 31.3 (5/16) |
| Diabetes | 5.9 (1/17) | 0.0 (0/17) | 0.0 (0/16) | 0.0 (0/16) |
| LDL-C (mg/dL), mean ± SD (N) | 130.4 ± 29.3 (17) | 125.9 ± 36.5 (17) | 113.8 ± 29.5 (15) | 107.6 ± 17.1 (16) |
| HDL-C (mg/dL), mean ± SD (N) | 55.4 ± 13.3 (17) | 56.1 ± 13.7 (17) | 49.7 ± 10.6 (15) | 53.1 ± 13.0 (16) |
| Fasting glucose (mg/dL), mean ± SD (N) ^#^ | 90.5 ± 15.1 (17) | 81.6 ± 8.4 (16) | 91.4 ± 12.2 (16) | 90.7 ± 12.1 (15) |
| HbA1c (%), mean ± SD (N) | 5.6 ± 0.4 (17) | 5.5 ± 0.3 (16) | 5.6 ± 0.3 (16) | 5.5 ± 0.3 (15) |
| Triglycerides (mg/dL), mean ± SD (N) | 109.2 ± 50.1 (17) | 90.1 ± 32.6 (17) | 105.2 ± 48.3 (15) | 93.1 ± 28.2 (16) |
| HOMA-IR, mean ± SD (N) | 3.2 ± 2.2 (17) | 1.4 ± 1.0 (16) | 2.5 ± 1.5 (16) | 1.9 ± 1.2 (15) |
| hs-CRP (mg/L), mean ± SD (N) | 3.1 ± 2.4 (17) | 3.0 ± 2.5 (17) | 2.8 ± 2.1 (15) | 3.1 ± 2.4 (16) |
| SBP (mmHg), mean ± SD (N) | 126.8 ± 14.8 (17) | 121.1 ± 13.6 (17) | 120.9 ± 12.6 (16) | 118.2 ± 11.2 (16) |
| DBP (mmHg), mean ± SD (N) | 76.7 ± 11.8 (17) | 73.9 ± 10.2 (17) | 79.6 ± 11.3 (16) | 75.6 ± 9.3 (16) |
| Total score for IWQOL-Lite-CT, mean ± SD (N) | 58.0 ± 17.1 (17) | 71.6 ± 15.6 (17) | 51.9 ± 14.9 (16) | 61.8 ± 17.2 (16) |
| ^#^ significant differences between the Epitome group and the placebo group at entry to the ELECT study (i.e. RESET week 24)  Abbreviations: BMI, body mass index; CI, confidence interval; DBP, diastolic blood pressure; HbA1c, glycated hemoglobin; HDL, high-density lipoprotein; IWQOL, Impact of Weight on Quality of Life-Lite-Clinical Trials; LDL, low-density lipoprotein; SD, standard deviation; SBP, systolic blood pressure. | | | | |

**TABLE S3: Summary of additional characteristics at baseline and week 24.**
